# 202. Targeting *Clostridioides difficile* Infection Prevention Efforts with Artificial Intelligence

**DOI:** 10.1093/ofid/ofae631.060

**Published:** 2025-01-29

**Authors:** Shengpu Tang, Rebekah Clark, Stephanie Shepard, Erkin Ötleş, Maxim Garifullin, Melinda Seiler, Justin Ortwine, Patrick Arnold, Jerod Nagel, Jeremy Jared, Sarah Krein, Jacob Kurlander, Paul Grant, Ji Baang, Anastasia Wasylyshyn, Krishna Rao, Jenna Wiens

**Affiliations:** Emory University, Atlanta, GA; University of Michigan, Los Angeles, California; University of Michigan, Los Angeles, California; University of Michigan - Michigan Medicine, Ann Arbor, Michigan; University of Michigan - Michigan Medicine, Ann Arbor, Michigan; University of Michigan - Michigan Medicine, Ann Arbor, Michigan; University of Michigan, Los Angeles, California; University of Michigan, Los Angeles, California; Michigan Medicine, Ann Arbor, Michigan; University of Michigan - Michigan Medicine, Ann Arbor, Michigan; VA Ann Arbor Healthcare System and University of Michigan, Ann Arbor, MI; University of Michigan - Michigan Medicine, Ann Arbor, Michigan; University of Michigan - Michigan Medicine, Ann Arbor, Michigan; Michigan Medicine, Ann Arbor, Michigan; University of Michigan, Los Angeles, California; University of Michigan, Los Angeles, California; University of Michigan, Los Angeles, California

## Abstract

**Background:**

Infections with *Clostridioides difficile* are associated with prolonged hospital stays, higher costs, and significant morbidity. Artificial intelligence (AI) tools can accurately predict which hospitalized patients are most likely to acquire *C. difficile* infection (CDI). However, to date, such tools have not been used in clinical practice. We investigated how AI tools for CDI risk stratification could be integrated into clinical workflows to promote targeted infection prevention efforts.

Details of the infection prevention bundle

**Figure:**
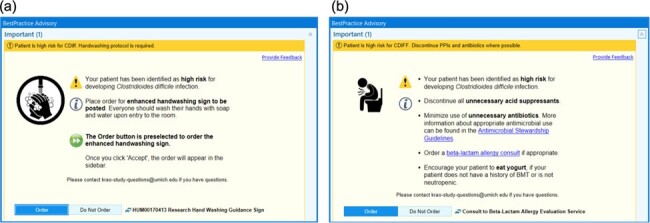

(a) Screenshot of the BPA for enhanced handwashing precautions. This BPA instructs the receiving provider to place an order for putting up the “Enhanced Handwashing Precautions” sign, depicted in Figure 2. (b) Screenshot of the BPA for antimicrobial stewardship. This BPA is educational and provides a list of recommendations for reducing risk of CDI, including discontinuing unnecessary acid suppressants, minimizing unnecessary antibiotics, consulting the beta-lactam allergy evaluation service, and encouraging patient to eat yogurt if appropriate.

**Methods:**

A previously validated AI model for predicting CDI risk from routinely collected data in electronic health records was used to generate daily risk scores for adult inpatients presenting to Michigan Medicine between January 1, 2023 and December 31, 2023. These scores were used to focus infection prevention efforts on high-risk patients in 10 selected hospital units with the greatest concentration of CDI cases. The infection prevention bundle, aimed at reducing both susceptibility and exposure, included provider-facing best practice alerts (BPAs) for enhanced handwashing precautions and antimicrobial stewardship (**Figure 1**). Using retrospective data, we determined a risk threshold that targets 5 alerts/unit/week on average. Clinical staff on selected units were educated about the AI tool by the study team.

Picture of the “Enhanced Handwashing Precautions” sign

**Figure:**
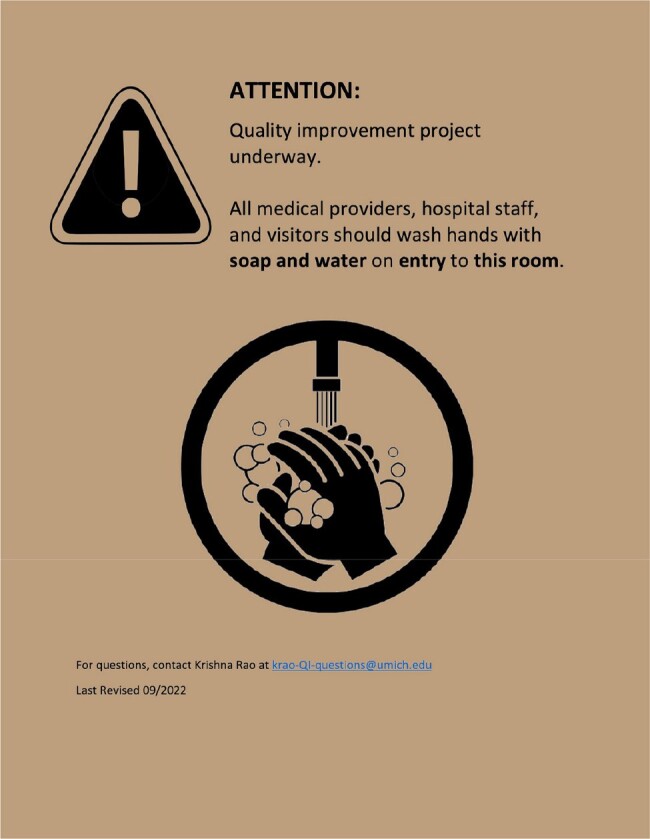

This sign is placed on the door of the rooms for high-risk patients in selected hospital units and instructs all persons to wash their hands with soap and water upon room entry.

**Results:**

During the study, 12,983 hospitalizations corresponding to 10,815 patients were assessed daily by the model, totaling 109,068 CDI risk scores. Among this population, 2,151 (16.6%) high-risk hospitalizations exceeded the risk threshold and triggered BPAs (an average of 4.1 alerts/unit/week). Among the high-risk population, 1,647 (76.6%) and 117 (5.4%) hospitalizations received an order for enhanced handwashing precautions and an order for a β-lactam allergy evaluation consultation, respectively. Field observations and interviews with clinical staff revealed challenges associated with behavior changes such as compliance with handwashing using soap and water to remove spores.

**Conclusion:**

AI tools can be integrated into clinical workflows to promote targeted infection prevention efforts. However, continuous monitoring of how such tools interact with existing workflows and education on novel infection prevention strategies are key to success.

**Disclosures:**

**Krishna Rao, MD, MS**, Merck and Company, Inc.: Grant/Research Support|Rebiotix Inc.: Advisor/Consultant|Seres Therapeutics: Advisor/Consultant|Summit pharmaceuticals: Advisor/Consultant

